# An aptasensor for the detection of *Mycobacterium tuberculosis* secreted immunogenic protein MPT64 in clinical samples towards tuberculosis detection

**DOI:** 10.1038/s41598-019-52685-6

**Published:** 2019-11-07

**Authors:** Marzhan Sypabekova, Kanat Dukenbayev, Anna Tsepke, Akmaral Akisheva, Nurlan Oralbayev, Damira Kanayeva

**Affiliations:** 1 Graduate Program in Science, Engineering, and Technology, Nazarbayev University, Nur-Sultan, 010000 Kazakhstan; 2 National Laboratory Astana, Nazarbayev University, Nur-Sultan, 010000 Kazakhstan; 3 Department of Electrical and Computer Engineering, School of Engineering and Digital Sciences, Nazarbayev University, Nur-Sultan, 010000 Kazakhstan; 4 Astana Antituberculosis Dispensary, Nur-Sultan, Kazakhstan; 5 Department of Biology, School of Sciences and Humanities, Nazarbayev University, Nur-Sultan, 010000 Kazakhstan

**Keywords:** Oligonucleotide probes, Diagnostic markers, Infectious-disease diagnostics

## Abstract

This work presents experimental results on detection of *Mycobacterium tuberculosis* secreted protein MPT64 using an interdigitated electrode (IDE) which acts as a platform for capturing an immunogenic protein and an electrochemical impedance spectroscopy (EIS) as a detection technique. The assay involves a special receptor, single stranded DNA (ssDNA) aptamer, which specifically recognizes MPT64 protein. The ssDNA immobilization on IDE was based on a co-adsorbent immobilization at an optimized ratio of a 1/100 HS-(CH_6_)_6_-OP(O)_2_O-(CH_2_CH_2_O)_6_-5′-TTTTT-aptamer-3′/6-mercaptohexanol. The optimal sample incubation time required for a signal generation on an aptamer modified IDE was found to be at a range of 15–20 min. Atomic Force Microscopy (AFM) results confirmed a possible formation of an aptamer - MPT64 complex with a 20 nm roughness on the IDE surface *vs*. 4.5 nm roughness for the IDE modified with the aptamer only. A limit of detection for the EIS aptasensor based on an IDE for the detection of MPT64 in measurement buffer was 4.1 fM. The developed EIS aptasensor was evaluated on both serum and sputum clinical samples from the same TB (−) and TB (+) patients having a specificity and sensitivity for the sputum sample analysis 100% and 76.47%, respectively, and for the serum sample analysis 100% and 88.24%, respectively. The developed aptasensor presents a sensitive method for the TB diagnosis with the fast detection time.

## Introduction

Tuberculosis (TB) is the leading cause of death from a single infectious agent accounting for over 1.33 million deaths in 2017 of the total 10 million infected people^1^. The distribution of TB epidemics varies by country with the most prevalence being in Africa (72%), India (27%), China (9%), Indonesia (8%), and Philippines (6%)^2^. The complication of TB control is also affected by the continuing emergence of drug resistant strains in countries such as India (21%), China (13%), and Russian Federation (10%) and countries of the former Soviet Union (8.5%)^1^. Moreover the 23% of the world’s population are estimated to have a latent TB infection, being at higher risk of developing TB during their life time^1,2^.

Technological breakthrough and intensified research are the priority targets of the end TB Strategy 2030 by World Health Organization (WHO) in fields of rapid diagnostics for the use at the point of care^1^. Current TB diagnostics tests include a DNA amplification test, sputum smear microscopy, and culture-based methods^3^. The DNA amplification test recommended by WHO is the Xpert MTB/RIF assay (Cepheid, USA) which targets the diagnosis of TB that can provide results within two hours^1^. The test is highly sensitive, however, it can identify the genetic information of both viable and dead *M*. *tuberculosis* with high precision and accuracy^4^. Moreover, since the test is not so cheap, in resource-limited countries, where TB is at the highest rate, such tests are performed only for specific and special cases. Sputum smear microscopy is a common diagnostic test, which involves an examination of the sputum sample under the microscope for the presence of *M*. *tuberculosis*. The technique is straightforward, however, less sensitive with the possibility of providing false-negative results. Culture-based methods involve the growth of *M*. *tuberculosis* in special conditions in BSL-3 laboratories for at least 2–8 weeks for obtaining the results extending the diagnosis time.

The level of expression of the MPT64 protein is high in individuals with an active TB. The main function of the protein is to promote survival and persistence in the host cell. It deactivates the expression of apoptotic cytokines, hence, plays a major role in survival and virulence of the mycobacteria^5^. MPT64 is expressed and secreted by the actively dividing *M*. *tuberculosis* along with other 33 predominantly secreted proteins by MTBC^6–8^. Such secreted proteins, also called antigens, are recognized by immune cells and initiate the first host immune response. The MPT64 protein can be found both in sputum and serum exosomes of a TB infected patient^9,10^.

This work presents an alternative way of diagnosing an active TB in sputum and serum clinical samples. The test involves a special receptor, single stranded DNA (ssDNA) aptamer, immobilized on an interdigitated microelectrode (IDE), which acts as a platform for capturing an immunogenic protein MPT64. The ssDNA aptamer used in the assay had a sequence of 40 nucleotides and was selected by Systematic Evolution of Ligand by Exponential Enrichment (SELEX) method against MPT64^11,12^. MPT64 ssDNA aptamer had a modified structure in the form of HS-(CH_2_)_6_-OP(O)_2_O-(CH_2_CH_2_O)_6_-5′-TTTTT-aptamer-3′. The surface chemistry was based on a simple and straightforward procedure of co-immobilization of an aptamer and 6-mercaptohexanol (MCH). The overall detection time of the assay was reduced from several hours down to 15 min, and the sensitivity was increased up to a fM range as compared to previously published results^13^. The surface chemistry used in the assay for improved MPT64 detection was based on a co-adsorbent immobilization at an optimized ratio of 1/100 ssDNA aptamer/MCH^13^. The surface chemistry used in this study was based on simultaneous immobilization of both aptamer and co-adsorbent in the form of MCH, and, hence, the procedure is simple with minimal cost.

## Results

### Aptasensor optimization studies

**Figure 1:**
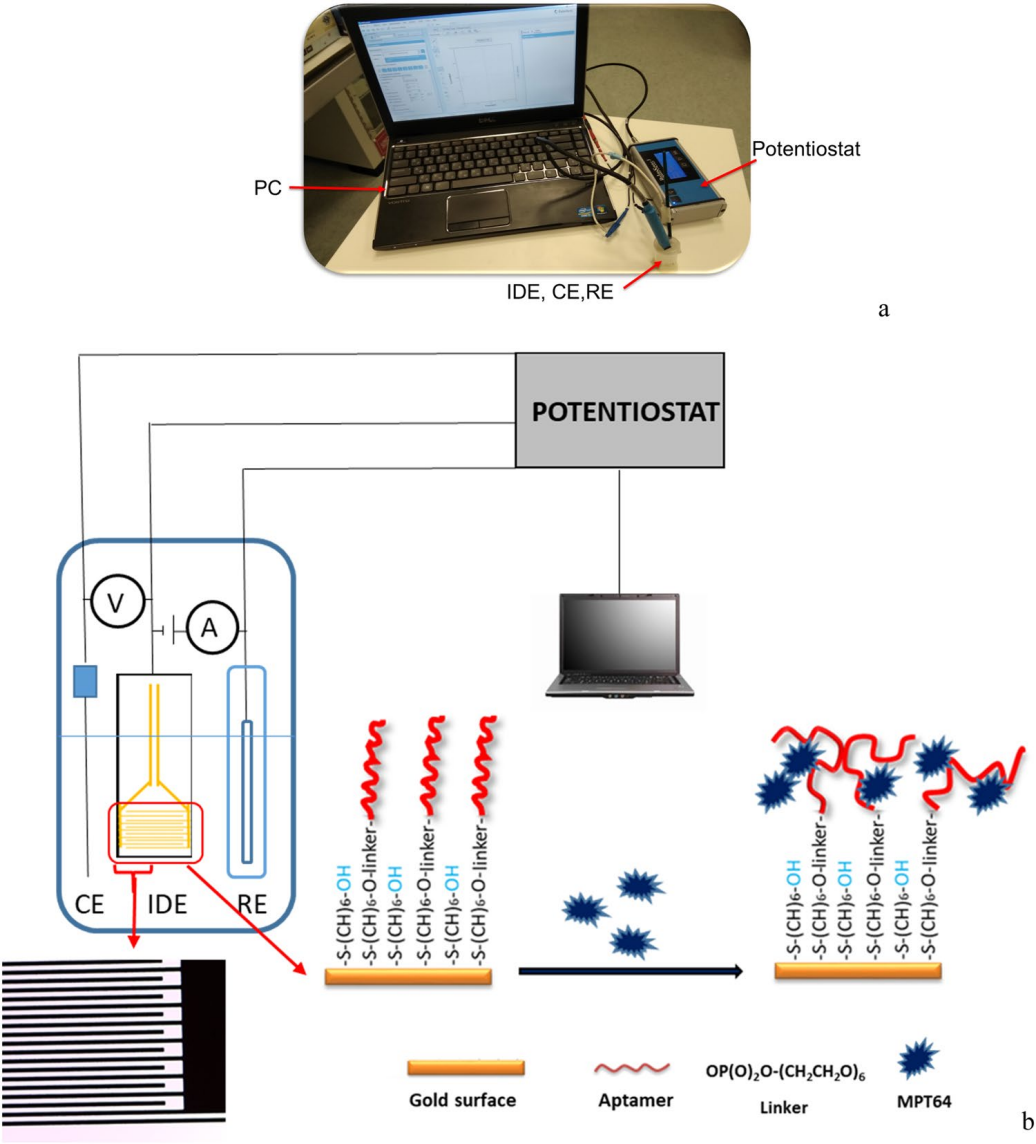
An experimental set-up (**a**) real, and (**b**) schematic comprising a PC and electrodes (IDE, CE, RE) that were connected to the portable potentiostat. The active area of the IDE indicated by a red rectangle composed of two interdigitated electrodes with two connection tracks, all made of gold, on a glass substrate with bands/gaps at 5 µm with dimensions L 22.8 × W 7.6 × H 0.7 mm. The part of the active area was magnified under the microscope. The gold coated surface of the IDE fingers was modified with the aptamer/MCH complex at a ratio of 1/100. The detection was based on the binding event of the target MPT64 onto the aptamer modified electrode surface.

**Figure 2:**
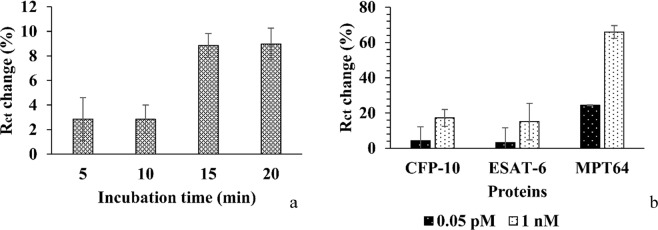
(**a**) An optimization study of the incubation time (5, 10, 15, and 20 min) required for a 1 fM MPT64 detection with the use of the electrochemical aptasensor and aptamer/MCH modified IDE. (**b**) A specificity study of the electrochemical aptasensor for the target MPT64 detection along with CFP-10 and ESAT-6 at different concentrations based on the DNA aptamer/MCH surface chemistry at an optimized ratio of 1/100.

**Figure 3:**
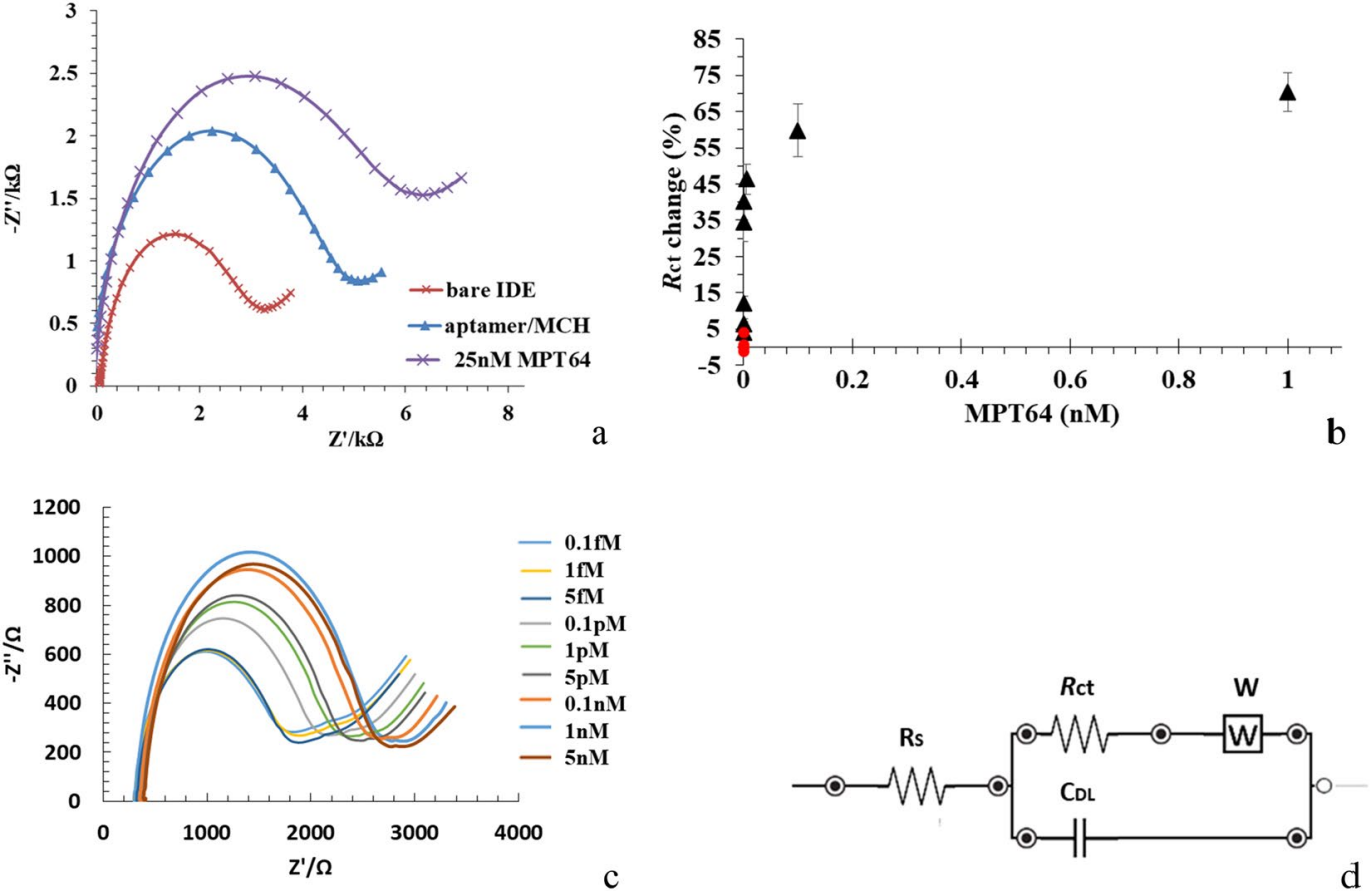
(**a**) The representative Nyquist plot (−Z″ *vs*. Z′) of bare IDE, after an aptamer/MCH immobilization step and a 25 nM MPT64 detection in the frequency range from 0.1 Hz to 50 MHz with a 10 mV a.c. The measurements were recorded in SELEX buffer containing 2 mM ferro/ferricyanide [Fe(CN)_6_]^3−/4−^ redox couple. (**b**) A concentration-dependent curve for different MPT64 concentrations using aptamer/MCH at a ratio of 1/100 relative to blank measurements from the EIS signal measured in SELEX buffer. (**c**) Experimentally obtained Nyquist plot for binding to MPT64 at concentrations from 0.1 fM to 5 nM. (**d**) Randles equivalent circuit, where *R*_s_ is the solution resistance, *R*_ct_ is the charge transfer resistance, *C*_DL_ is the double layer capacitance, and *W* is the Warburg element.

The overall set-up of the aptasensor is presented in Fig. 1. It consists of a portable potentiostat that is connected to the modified IDE with an aptamer/MCH complex at a ratio of 1/100 and a portable computer. An optimization study of the target protein incubation time was performed, where a 1fM MPT64 was incubated on the modified IDE at time points of 5, 10, 15, and 20 min (Fig. 2a). The IDE surface was modified by the aptamer/MCH complex at a ratio of 1/100, and the EIS signal was measured in the SELEX buffer (50 mM Tris-HCl; 25 mM NaCl; 5 mM MgCl_2_; pH 7.5). After incubation with MPT64 protein, IDE was rinsed with the measurement buffer, and EIS was recorded in the measurement buffer with 2 mM ferro/ferricyanide [Fe(CN)_6_]^3−/4−^ redox couple. The EIS signal was fitted with the equivalent Randles circuit (Fig. 3d) and charge transfer resistance *R*_ct_ with an error value below 2% was used to report the results. The change in charge transfer resistance (*R*_ct_) was calculated by subtracting the *R*_ct_ value of an electrode without protein from the electrode incubated with protein at respective incubation times. The results show that at 5, 10, 15, and 20 min protein incubation time, the average change in *R*_ct_ value was 2.85, 2.83, 8.85, and 8.98%, respectively (Fig. 2a). The statistically significant difference between the 5–10 min incubation time and 15–20 min incubation based on one-way analysis of variance presented P value as 0.0004 which was below 0.05 indicating the significant statistical difference between the two incubation timing sets.

### Specificity test

The aptasensor was tested for its specificity against other non-target proteins such as ESAT-6 and CFP-10. The aptasensor, measured with redox markers in a solution, showed a *R*_ct_ signal change that increased by 65.98% at 1 nM and 24.48% at 0.05 pM when it was incubated with MPT64 protein, whereas, it remained less than 18% at 1 nM and 5% at 0.05 pM for the control proteins ESAT-6 and CFP-10 (Fig. 2b). Change in *R*_ct_ of the IDE was measured after 15 min protein incubation followed by rinsing and measurement in SELEX buffer (50 mM Tris-HCl; 25 mM NaCl; 5 mM MgCl_2_; pH 7.5) containing 2 mM ferro/ferricyanide [Fe(CN)_6_]^3−/4−^ redox couple. The statistically significant difference between the binding to MPT64, ESAT-6, and CFP-10 based on one way analysis of variance showed that at a 0.05 pM protein concentration the P value was 0.09 and for a 1 nM protein concentration the P value was 0.005 which indicates that the statistical difference for the later concentration was significantly different.

### Concentration-dependent analysis

The charge transfer resistance *R*_ct_ of the clean bare IDE was 2.714% (Fig. 3a and Table 1). The electrode surface was modified by immobilizing an aptamer/co-adsorbent complex as described in aptamer/MCH immobilization on the IDE Section. After incubation with the aptamer/MCH, *R*_ct_ increased to 4.164% as the DNA aptamer probe is negatively charged and, therefore, inhibited electrons from the mediator reaching the electrode^14^. Incubation with the 25 nM MPT64 caused a further increase in *R*_ct_ change up to 4.734%.

**Table 1:** The *R*_ct_ change of the bare IDE, aptamer/MCH modified IDE and after 25 nM MPT64 binding.

Step	*∆R*_ct_ (%)
Bare IDE	2.714
Aptamer/MCH	4.164
After 25 nM MPT64 binding	4.734

A concentration-dependent curve for different MPT64 concentrations relative to blank measurements from the EIS signal measured in SELEX buffer (50 mM Tris-HCl; 25 mM NaCl; 5 mM MgCl_2_; pH 7.5) is presented in Fig. 3b. A concentration-dependent analysis was performed for MPT64 concentrations ranging from 0.1 fM to 1 nM and showed a corresponding increase in *R*_ct_. Figure 3d shows the Randles equivalent circuit model selected to fit the experimental data, in which *R*_s_ is the solution resistance connected in series with the double layer capacitance *C*_DL_ and in parallel with the charge transfer resistance *R*_ct_ of the surface and Warburg element *W* to model diffusion. A concentration-dependent response was obtained with a gradual increase in *R*_ct_ signal up to 1 nM MPT64 demonstrating a good response down to concentration of 1 fM with the limit of detection (LoD) of 4.1 fM for MPT64 detection. The lowest concentration that was tested for MPT64 was 0.1 fM. The LoD was calculated using the formula LoD = mean _blank_ + 1.645(SD_blank_) + 1.645(SD_low concentration sample_), where SD is the standard deviation^15,16^. The signal for SELEX buffer with the target MPT64 protein alone remained unchanged throughout the experiment (Fig. 3b, red round dots). The surface of the sensor did not saturate at 1 nM of MPT64. Figure 3c presents an experimental Nyquist plot for binding of MPT64 at concentrations ranging from 0.1 fM to 5 nM. A fitting circuit was used in this study to quantify the target analyte.

### Surface characterization studies with an AFM imaging

**Figure 4:**
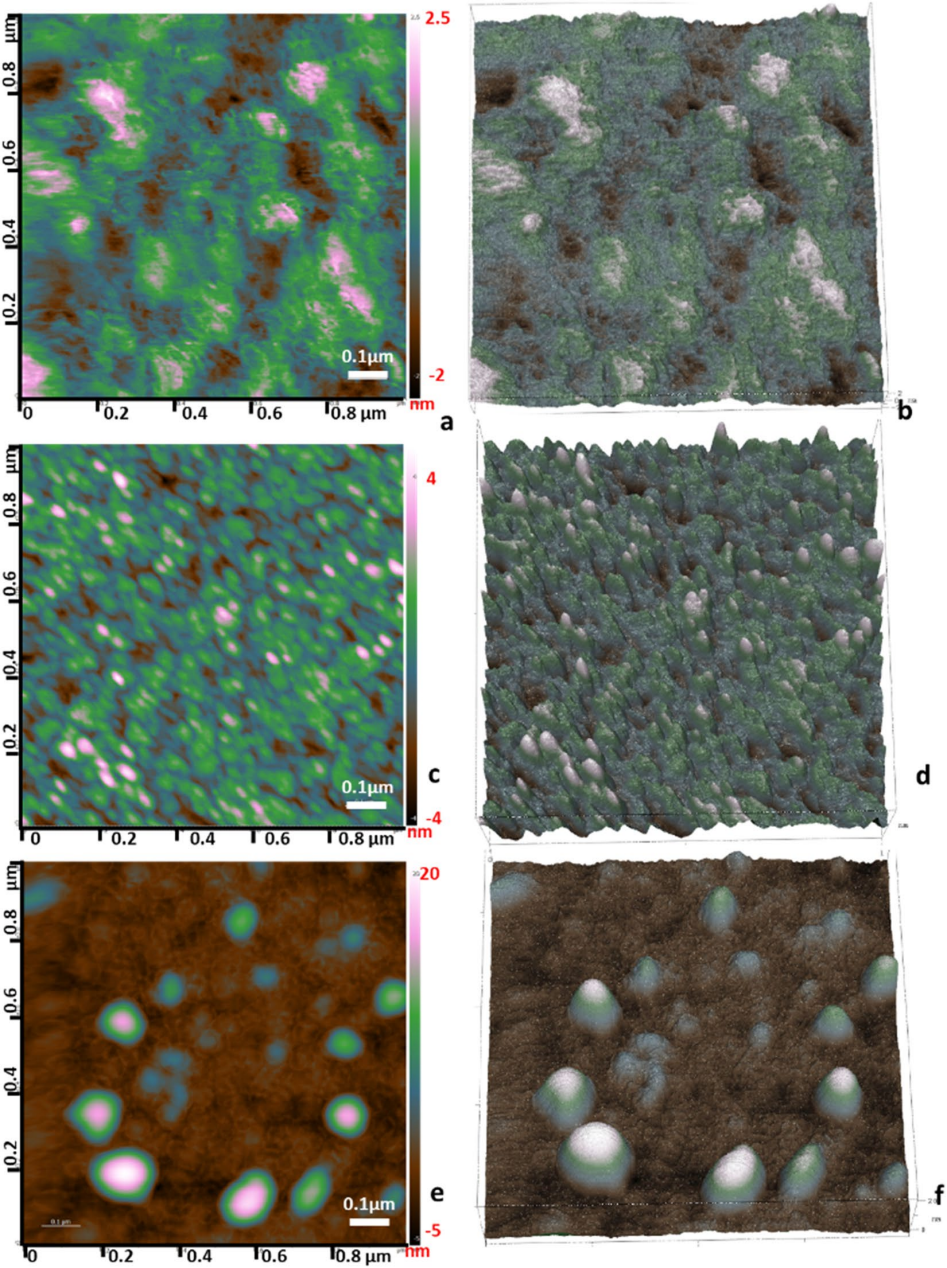
AFM topographical micrographs in air of G-IDEAU 05 type gold plated IDE surfaces. (**a**,**b**) 2D and 3D micrographs of a bare IDE with the surface roughness of around 2.6 nm. (**c**,**d**) 2D and 3D micrographs of the IDE surface functionalized with DNA aptamer/MCH complexes with an average roughness of the IDE surface of 4–4.5 nm high. (**e**,**f**) 2D and 3D micrographs of the aptamer functionalized IDE surface that captured a 1 nM MPT64 with the IDE surface roughness of up to 20 nm high.

Bare IDE was cleaned as indicated in the Section Electrode cleaning. AFM imaging showed that the surface roughness of a clean IDE was around at 2.6 nm (Fig. 4a,b). The IDE surface was further functionalized with DNA aptamer/MCH complexes that had the average roughness of the IDE increased up to 4–4.5 nm as compared to the bare IDE indicating a successful immobilization of aptamers (Fig. 4c,d). The aptamer size used in this study was relatively small (40 nucleotides), and its single-stranded nature made them fold into favourable shape and, hence, appeared as a small globular structure under the AFM. The IDE was then incubated with a 1 nM MPT64 for 15–20 min followed by rinsing to remove any unbound material from the surface and consequent drying under the gentle stream of N_2_. It can be seen from Fig. 4e,f that there was MPT64 protein captured on the IDE surface that potentially formed protein-protein complexes as the surface roughness of the IDE increased up to 20 nm, and a topography of the IDE surface showed large globular structures.

### Clinical sample analysis

**Figure 5:**
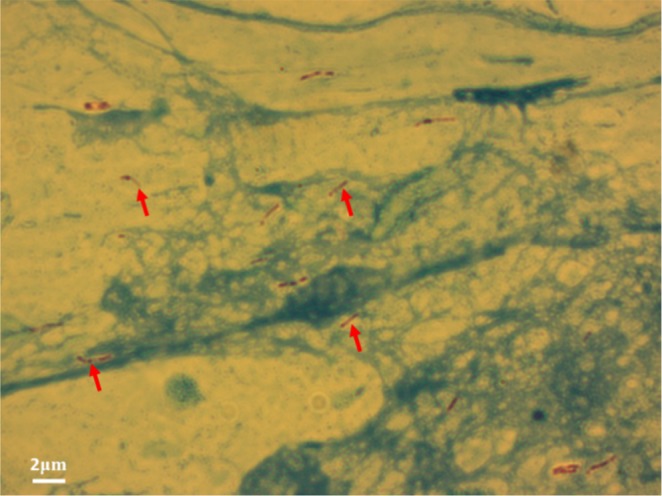
A representative micrograph of *M*. *tuberculosis* bacilli using an AFB staining technique from a sputum sample of a TB infected patient. Arrows indicate stained rod-shaped bacilli corresponding to the presumptive *M*. *tuberculosis*.

Clinical samples were tested to further evaluate performance of the developed aptasensor. Overall, 17 TB (+) and 4 TB (−) clinical samples were evaluated. Both serum and sputum samples were analyzed from the same patient to observe the performance of the MPT64 aptasensor on a type of sample. Hospital personnel collected the sputum and serum samples from patients in special containers at anti-Tuberculosis dispensary (Nur-Sultan, Kazakhstan). All samples were confirmed for the presence of *M*. *tuberculosis* in TB infected patient samples using an AFB staining microscopy (Fig. 5). Samples from four TB (−) individuals were used as a control.

**Figure 6:**
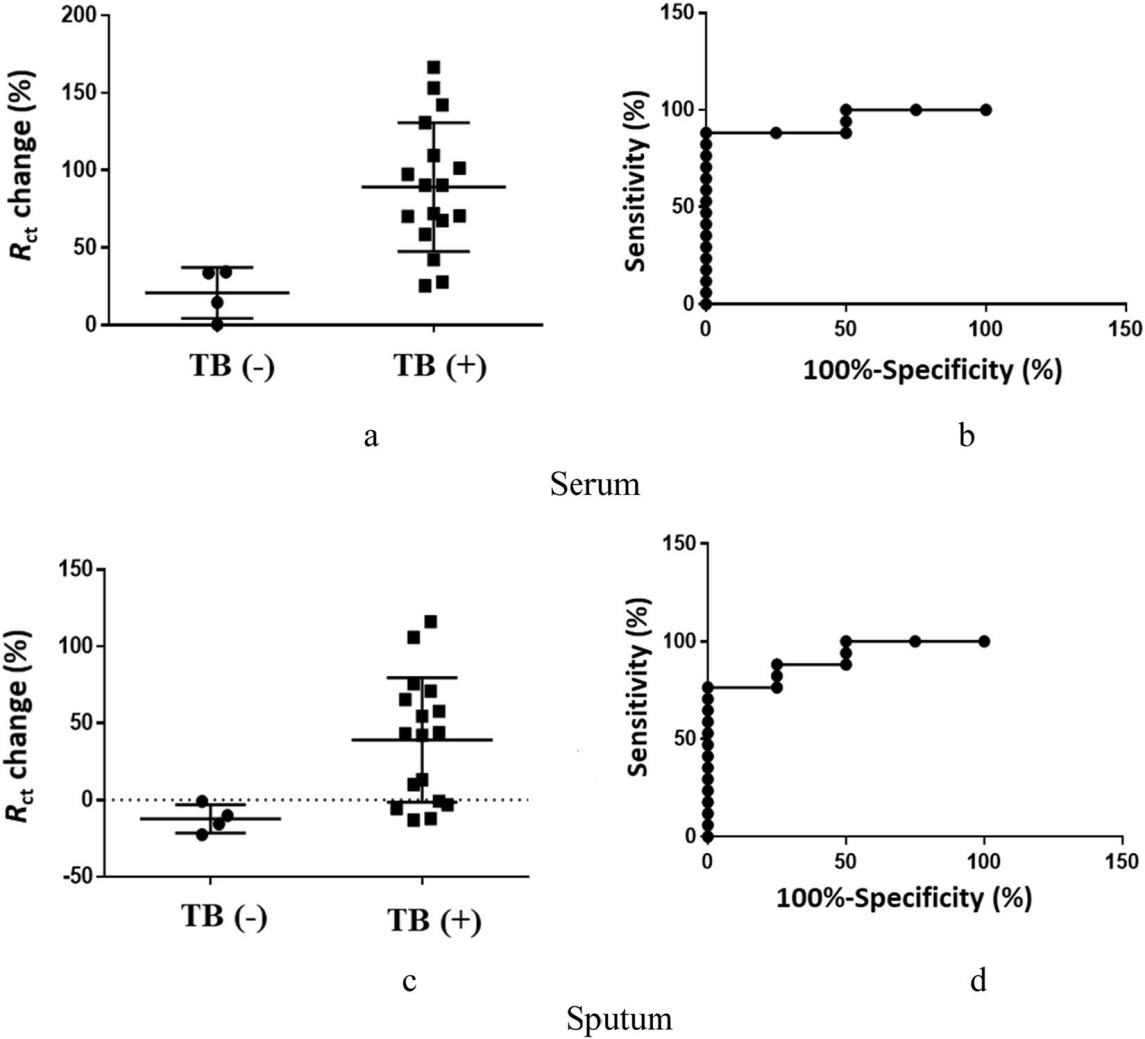
Evaluation of the aptasensor using clinical (**a**) serum and (**c**) sputum samples from infected TB (+) (active form) and TB (−) individuals and respective ROC curves (**b**,**d**).

As it can be seen from Fig. 6a,c, the general trend shows that there was a higher change in *R*_ct_ signal when the electrode was incubated with TB (+) patient samples. The *R*_ct_ for TB (+) serum sample analysis increased from 25.44% up to 166.58%, it remained less than 34.47% for TB (−) samples (Fig. 6a). The *R*_ct_ for TB (+) sputum sample analysis varied from −13.14% to 116.04 and for TB (−) samples varied from −0.92% to −22.53% (Fig. 6c). The dotted line in Fig. 6c was placed to separate the positive and negative *R*_ct_ values. The specificity and sensitivity of the sputum sample analysis were calculated as 100% (95 CI: 50.1–93.19%) and 76.47% (95% CI: 39.76–100.0%), respectively, using Youden’s index. The area under the curve (AUC) for sputum samples was 91.18% (95% CI: 73.51–100%) from the ROC curve (Fig. 6d). Specificity and sensitivity of the serum sample analysis were 100% (95% CI: 39.76–100.0%) and 88.24% (95% CI: 63.56–98.54%), respectively. The AUC for serum sample was 94% (95% CI: 80.6–100%) (Fig. 6b).

## Discussion

In this paper, we further evaluated the performance of the aptasensor for the detection of MPT64 in clinical samples. The immobilization of the aptamers based on the optimized surface chemistry^13^ was done on the surface of the IDE, and the EIS signal was recorded using the portable potentiostat. EIS is the technique used in impedimetric electrochemical biosensors. As compared to other biosensors, the impedimetric technique provides low cost, low power, scalable, and highly sensitive and label-free measurement with minimal hardware demand^17^. The EIS Faradaic mode was used within the scope of this work. It is based on transportation of redox species (ferro/ferricyanide) between the electrode and electrolyte solution and, hence, measures the resistance for electron transfer. The signal in this way can be recorded within a small change of analyte binding event based not only on molecular interaction level but also on electron/charge transfer levels^13^ hence provides sensitive detection down to attomolar range^18,19^.

IDE is a special platform with separated metal plates that have a number of digits overlapping with one another. Figure 1b represents a micrograph of the IDE with an active area to which the aptamer was immobilized. Prior surface modification, each IDE electrode was cleaned as described in the Electrode Cleaning Section. Cleaning of the IDE surface was an essential step in the aptasensor development as it removed physically adsorbed random molecules and any dust from the IDE surface. The application of an electric signal onto the IDE created an electric field between the digits. When a specific molecule (e.g. protein, DNA, cell) immobilized on the digits, the electric field was altered or disrupted resulting in the change of electric field. The geometry of the IDE (digit width and spacing) played an important role in the aptasensor sensitivity. The sensitivity of the sensor increases with a decreasing distance between the digits^20,21^. Therefore, analytes with different sizes (bacteria^22^, virus^23^, protein^24^, DNA^25^) could be detected using the IDE with different interdigit spacing (100–250 µm, 15 µm, 5–10 µm, 150 nm, respectively). In general, IDEs have a relatively small dimension, which makes them suitable to integrate with a miniaturized device as well as to analyze a small sample volume in the absence of interference with selectivity and response time. In this study, for the detection of MPT64 protein, we used the IDE with the interdigit spacing of 5 µm for the aptamer immobilization. It should be noted that Fig. 3a, represents the impedance spectra for modification steps for one particular electrode. The general trend in decrease/increase of *R*_ct_ change before and after cleaning, after aptamer/MCH complex immobilization and protein incubation for other electrodes, was similar.

The LoD of the sensor was increased up to 4 fM in the measurement buffer, showing the improved sensitivity of using the IDE for MPT64 detection as compared to the LoD 81 pM using a plane gold electrode as an immobilization platform^13^. An increase in charge transfer resistance (*R*_ct_) was observed with an increasing MPT64 concentrations from 0.1 fM to 1 nM (Fig. 3b). A 40 nucleotide long DNA aptamer with a long linker in the form of HS-(CH_2_)_6_-OP(O)_2_O-(CH_2_CH_2_O)_6_-TTTTT-aptamer (Fig. 1) was used in the study. The function of the thymine residues was to provide spacing for improved interaction with the protein as well as eliminate the direct adsorption of the aptamer onto the electrode surface. The long linker used in this study was based on a hydrophilic ethylene glycol (CH_2_CH_2_O)_6_ group which enabled the aptamer sequence to protrude from the modified electrode surface. Ethylene glycol was also used to eliminate any steric hindrance associated with the binding process as well as to reduce a non-specific interaction^26,27^.

The AFM micrographs revealed the formation of a complex between the immobilized aptamer and the MPT64 protein, where the surface roughness of the IDE surface was increased from 4.5 nm up to 20 nm after protein incubation (Fig. 4a–f). The shape of the complexes as well as the variations in height from 5–20 nm is indicative of non-uniform IDE surface coverage after protein incubation. The relationship between the surface roughness measured by AFM (2.6 nm; 4–4.5 nm; 20 nm, respectively) and a change in *R*_ct_ signal detected by EIS (2.714%; 4.164%; 7.734%) for a bare electrode, an aptamer/MCH functionalized electrode, and an electrode capturing the target MPT64 had a correlation in values except for the value in the aptamer/MCH functionalized electrode. Although two methods were based on different detection methods, one was measured in a solution (EIS) and the other was measured in air (AFM) there more experiments could be done since AFM cannot fully provide adsorption properties of the complex formation.

The incubation time optimization studies showed that time required for protein binding at 5 min and 10 min had on average relatively large error bars in comparison to 15 min and 20 min incubation time (Fig. 2a). This inconsistency could be due to the instability of the target protein on the surface as well as indicative of large signal variability from one electrode to another. The change in *R*_ct_ value was increased three times higher at 15 min and 20 min (Fig. 2a) as compared to 5–10 min incubation time. The increase in *R*_ct_ signal after 15 min incubation time could correspond to protein stabilization and specific recognition by the aptamers immobilized on the surface of the IDE. It was concluded that 15–20 min sample incubation time on IDE modified surface is optimal for recording the EIS signal and, hence, reducing the overall detection time.

The aptasensor showed specificity towards MPT64 as compared to other *M*. *tuberculosis* secreted immunogenic proteins such as ESAT-6 and CFP-10. Perilous study with the same aptamer sequence showed that aptamer had very good specificity towards MPT64 protein as compared to other proteins such as PSA, CEA, and HSA^13^. HSA, CEA, and PSA are biomarkers that present in human serum and associated with other diseases such as cancer but not related to TB^28,29^. In the current study, we tested the developed aptasensor for its specificity towards *M*. *tuberculosis* secreted proteins, such as CFP-10 and ESAT-6 along with MPT64 (Fig. 2b). CFP-10 (10 kDa) and ESAT-6 (6 kDa) are proteins involved in *M*. *tuberculosis* virulence. They are one of the most abundant antigens of *M*. *tuberculosis* grown in the culture^30^. CFP-10 and ESAT-6 are encoded by genes aligned in pairs in the *M*. *tuberculosis* genome. The product of the genes is then secreted by specialized transport system^31,32^. ESAT-6 and CFP-10 form a tight one to one complex^31^. One of the main characteristics of this complex is that C-terminus of CFP-10 forms a long flexible arm, which plays an important role in a cell surface attachment specifically binding to macrophages and monocytes These proteins like MPT64 also stimulate the production of T-cells and are among candidate vaccines for TB^33^. As it can be seen from Fig. 2b the aptasensor is more specific towards the MPT64 protein as compared to ESAT-6 and CFP-10. The inconsistency in large error bars for the ESAT-6 and CFP-10 could be due to non-specific binding of the control proteins to the functionalized surface.

The current study also presents results on the potential application of the developed aptasensor using clinical samples. The conventional TB diagnostic technique Acid Fast Bacilli (AFB) staining microscopy uses the sputum sample for the identification of *M*. *tuberculosis*^1^. MPT64 protein is secreted by and expressed on the actively dividing *M*. *tuberculosis*^34^, therefore in this assay, the sputum sample was used to test the aptasensor for the presence of MPT64. Later studies showed the presence of MPT64 protein also in serum samples^10^. Therefore, in addition to the sputum sample in this study, serum sample was also used to evaluate the aptasensor for the presence of MPT64. ROC curve analysis was used for the evaluation of the diagnostic performance of the aptasensor, or the accuracy of the test to discriminate diseased cases from normal cases^29^. The ROC curve was plotted as the sensitivity of a function of the false positive rate for a specific cut-off point. The closer the ROC curve to the upper left corner the higher the accuracy of test^29^. The Youden index *J*, in this work, was defined as *J* = max (sensitivity + specificity − 1) and corresponded to the maximum vertical distance between the ROC curve and the diagonal line^35^. Equal weight was given to sensitivity and specificity while selecting optimal Youden index criterion. Sensitivity in this test was considered as a probability that a test result would have been positive when the disease was present (true positive), and it was expressed in a percentage. Specificity was a probability that a test result would have been negative when the disease was not present (true negative). The results clearly showed the more *R*_ct_ signal increase in TB positive samples with specificity and sensitivity for the sputum sample analysis being at 100% and 76.47% and for the serum sample being at 100% and 88.24%, respectively. The results clearly show that there are still further improvements needs to be done to elevate the sensitivity of the assay. The sensitivity of the assay is considered reliable, which is above 94–65%. The composition of the clinical sample is very complex, and therefore, a non-specific binding of un-related bio-molecules on to the surface is an issue that every aptasensor may face. For instance, the non-specific binding of the HSA protein onto the MCH modified surface accounted in the increase of *R*_ct_ signal by 4–6%^13^. It shows that, indeed, the signal from EIS could not be only due to the specific binding between aptamer and protein in interest but it could also account for the non-specific binding of an un-related biomolecules onto the electrode surface. In this instance, the signal to noise ratio needs to be carefully calibrated and set as well tuned surface chemistry to avoid a non-specific adsorption on the electrode surface. The preliminary results of this study showed that the aptasensor is capable of distinguishing TB (+) from TB (−) samples. However, we should mention that the threshold needs to be set to clearly identify the presence of MPT64.

Nevertheless, presented results showed that the IDE based aptasensor for the detection of *M*. *tuberculosis* secreted protein MPT64 works quite well for the analysis of both type of clinical samples, sputum and serum, with an emphasis more towards the serum sample analysis. One advantage of the clinical TB serum sample analysis with the use of the developed aptasensor is a safer way of handling of the serum sample during diagnosis and sample utilization after the measurement that would not require availability of high level biosafety laboratory facilities as it is required for the TB sputum sample diagnosis. The developed aptasensor can be further evaluated using an increased number of clinical samples for the better and concrete prognosis of the test. In addition, the signal enhancement could be obtained by using a secondary aptamer conjugated with nanoparticles for better confirmation of the results between TB (+) and TB (−) samples.

## Materials and Methods

### Reagents

Thiolated MPT64 aptamer in the form of HS-(CH_6_)_6_-OP(O)_2_O-(CH_2_CH_2_O)_6_-5′-TTTTT-aptamer-3′ were synthesized by Eurogentec (Belgium)^11–13^. Magnesium chloride (MgCl_2_, ≥99%)_,_ sodium chloride (NaCl, ≥99.5%), sulfuric acid (H_2_SO_4_, 96%), potassium hexacyanoferrate (III), potassium hexacyanoferrate (II) trihydrate, and 6-mercapto-1-hexanol (MCH) used in the sample preparation were of analytical grade and purchased from Sigma-Aldrich, UK. Tris base (≥99.0%), isopropanol (propan-2-ol, HPLC grade) were purchased from Fisher Scientific. Absolute ethanol was purchased from Aidabul Distillery (Kazakhstan). Target immunogenic protein MPT64 (46 kDa) (Rv1980c) (Gene ID: 581375; Accession#: CAA53143) of *Mtb* H37Rv strain with a concentration of 1 mg/ml was obtained from EnoGene Biotech Co Ltd (Nanjing, China). His and Trx tagged MPT64 protein was purified from recombinant *Escherichia coli* strain by an affinity chromatography with a >90% purity. According to the manufacturer, the protein was suitable for the use in multiple immunoassay formats, including ELISA, Western Blot, and rapid tests. Non-target ESAT-6 (p463-1) and CFP-10 proteins were purchased from Sunny lab (USA). All aqueous solutions were prepared using 18.2 MΩcm ultra-pure water with a Pyrogard filter (Millipore, UK).

### Instrument

The data acquisition was performed with the Palmsens3 impedance analyzer (PalmSens BV, the Netherlands) with the PSTrace5 software. A three-electrode cell with an Ag/AgCl electrode (Ametek-AMT, Oak Ridge, TN, USA) and a platinum (Pt) counter electrode (Ametek-AMT, Oak Ridge, TN, USA) was used for all measurements. The tip of the Ag wire was coated with Ag/AgCl ink (ALS, Japan) and dried overnight at room temperature. The EIS was measured in a buffer containing 2 mM ferro/ferricyanide [Fe(CN)_6_]^3−/4−^ redox couple (potassium hexacyanoferrate II/III). The frequency range used for the measurement was in the range of 0.1 Hz to 50 MHz with 56 frequencies and with a 10 mV a.c. voltage superimposed on a bias d.c. voltage. All measurements were performed at room temperature inside a custom-made Faraday cage and corresponding signal was recorded in a Nyquist plot. The impedance change was calculated as the difference between the impedance measured before and after the protein was captured onto the IDE.

### Electrodes

An IDE (DRP-G-IDEAU5, Dropsens, Spain) was composed of two interdigitated electrodes with two connection tracks, all made of gold, on a glass substrate with bands/gaps at 5 µm with dimensions L 22.8 × W 7.6 × H 0.7 mm (Fig. 1b). Electrode consisted of 250 × 2 digits with digit length 6,760 µm. A cable connector (CACIDE, Dropsens, Spain) was used as an interface between an IDE and the potentiostat.

### IDE surface cleaning

Cleaning of an IDE was performed by sonicating in a solution containing propanol-2, and ethanol for 5 min followed by thorough rinsing and sonicating the IDE for 10 min in water. The IDE was then dried under the gentle stream of N_2_ until fully dry. Microscopy was used to observe any irregularities on the IDE surface. Electrodes with smooth geometry were used for further analysis. To characterize the effect of the cleaning procedure on the IDE, EIS was conducted using the Palmsens3 impedance analyzer before and after cleaning steps in a solution containing 2 mM of ferri/ferrocyanide ([Fe(CN)_6_]^3−/4−^) in the measurement buffer (SELEX buffer: 50 mM Tris-HCl; 25 mM NaCl; 5 mM MgCl_2_; pH 7.5).

### Aptamer/MCH immobilization on the IDE

A schematic overview of the IDE surface modification with an aptamer/MCH complex is presented in Fig. 1b. For the development of the MPT64 aptasensor, 50 µl mixture of a 100 µM of thiolated DNA aptamer and 100 µM of co-adsorbent at a ratio of 1/100 was dropped on a pre-cleaned IDE and was incubated for 16 h at 4 °C in a humid chamber. MCH was initially diluted in absolute ethanol at 100 mM to make a stock solution and was stored at −20 °C until further use. Further dilutions were prepared prior to incubation in the measurement buffer (SELEX buffer: 50 mM Tris-HCl; 25 mM NaCl; 5 mM MgCl_2_; pH 7.5). After incubation with the aptamer/MCH complex on the IDE, the electrode was rinsed with the measurement buffer to remove any unbound ssDNA aptamers. A 50 µl of 1 mM of MCH was applied onto the surface of the IDE for another 1 h at room temperature to ensure complete thiol coverage on the gold surface and to displace a non-specific interaction between the DNA and gold^36,37^. After rinsing the IDE with the measurement buffer, it was then placed into the measurement buffer with 2 mM ferro/ferricyanide [Fe(CN)_6_]^3−/4−^ redox couple for at least 1 h for stabilization purposes.

### Protein detection

The aptamer modified IDE was incubated in a solution containing target MPT64. A wide range of MPT64 concentrations diluted in the measurement buffer from 0.1 fM up to 1 nM were tested in this study. After incubation with MPT64 protein the electrode was rinsed with the buffer to remove any of the remaining unbound residues from the electrode surface. The electrode was then placed in a measurement buffer and signal was recorded. For the specificity studies the electrodes were incubated with the proteins ESAT-6 and CFP-10 for 15 min followed by rinsing and measurement in a buffer with 2 mM ferro/ferricyanide [Fe(CN)_6_]^3−/4−^ redox couple. A schematic overview of the aptasensor for the detection of MPT64 on the aptamer/MCH modified IDE surface is presented in Fig. 1b. All measurements were carried out at room temperature, in triplicates, and the mean value of replicates, standard deviations, and standard errors from the mean was used to report the results.

### Atomic force microscopy imaging

Atomic force microscopy (AFM) was conducted to further confirm the binding of the target MPT64 protein onto the DNA aptamer immobilized microelectrode surface. The share-force topographical measurements of the cleaned IDE (described in Section Electrode cleaning), aptamer modified IDE (described in Section Aptamer/MCH immobilization on the IDE), and IDE after MPT64 incubation (described in Section MPT64 detection) were scanned using an AFM system, AFM SmartSPM 1000 (AIST-NT, Russia). Prior to the AFM scanning, the IDE electrode was rinsed with dH_2_O and gently dried under the stream of N_2_. All images were produced using AC-Mode (non-contact or tapping mode) of operation. Corresponding AFM images were performed in air at ambient temperature. Scanning size of all images is in XY directions was 1,000 nm to 1,000 nm. The height Z directions were adjusted automatically during scanning for the whole period of scanning. Measurements scanning rate was set at 0.2 Hz with pixel resolution at 1,024 × 1,024. To visualize high-resolution images, Super Sharp type NSG30_SS cantilevers (Tips Nano) with tip radius curvature up to 5 nm and force constant value of 22–100 N/m were used. The resonance frequency of a cantilever was in a range of 200–440 kHz.

### Clinical sample analysis

Clinical samples were tested to further evaluate performance of the developed aptasensor on the detection of the target MPT64 protein. The aptasensor was tested on clinical samples obtained from TB patients based on serum and sputum samples. Analysis of samples was carried in a BSL-3 laboratory at the anti-tuberculosis dispensary, Nur-Sultan, Kazakhstan. The study was subject of exemptions from the ethical review of the Central (National) Ethics Commission (Ministry of Healthcare, Kazakhstan) and the Nazarbayev University research ethics committee. Written informed consents were received from the patients, and all samples were anonymized and coded. All methods were performed in accordance with relevant guidelines and regulations based on the criteria of Kazakhstani TB diagnostics standards. Overall, twenty-one sputum samples were screened, seventeen from patients with confirmed presence of *M*. *tuberculosis*, TB (+), and four from non-tuberculous controls, TB (−). Blood samples from patients were collected and centrifuged at 3,500 rpm for 10 min. The supernatant-serum was further used in the analysis. Sputum samples were collected in sterile containers from TB (+) and TB (−) patients. Collected sputum and serum samples were stored at 4 °C and further analyzed by the developed MPT64 aptasensor on the same day. Both sputum and serum samples were diluted in SELEX buffer (50 mM Tris-HCl; 25 mM NaCl; 5 mM MgCl_2_; pH 7.5) where aptamers were previously selected at a 1/10 ratio followed by thorough mixing. A 50 µl of each clinical sample was placed onto the IDE surface and allowed to be incubated for 20 min at room temperature. IDE was then rinsed with the SELEX buffer to remove any unbound biological material from the IDE surface that was then connected to a portable impedance analyzer (PalmSens BV, the Netherlands) via the cable connector (CACIDE, Dropsens, Spain) inside the custom-made Faraday cage.

### Acid fast bacilli (AFB) staining

AFB staining microscopy is a technique that examines the sputum samples using a microscope to determine the presence of *M*. *tuberculosis*. AFB staining in this study was used to visually confirm the presence of the *M*. *tuberculosis* within 17 TB (+) sputum samples. The AFB staining microscopy was performed in the BLS-3 laboratory at the anti-Tuberculosis dispensary following the standard microscopy procedure approved by WHO. A wooden applicator was used to transfer a very thin layer of a sputum from a cloudy, serous, yellowish region onto a clean microscope slide. The dimension of the layer that was deposited on a slide was 1–2 cm. Then, the slide was air dried for 15 min, which was then heat fixed under the flame 3–4 times without overheating the slide. Next, the slide was flooded with carbol fuchsin, which was then heated to dry and rinsed off under tap water. The slide was then flooded with a 1% solution of hydrochloric acid in isopropyl alcohol (or methanol) to remove the carbol fuchsin, thus, removing the stain from cells that were unprotected by a waxy lipid layer. Thereafter, cells were stained with methylene blue, and slides with presumptive *Mycobacterium* cells were visualized under a microscope (Zeiss Axio Observer, Zeiss Germany) with an immersion oil. The presence of rod-shaped bacilli on the stained microscope slide as shown in Fig. 5 was regarded as TB (+) sample.

### Data analyses

The diagnostic value of the assay using aptamers was evaluated using Receiver Operating Characteristics (ROC) curve analysis (GraphPad Prism 6, Graphpad Software, Inc). *R*_ct_ change values for the EIS assay were plotted and the area under the curves (AUC) and 99% confidence intervals (95% CIs) were calculated. The optimal cut-points were determined based on the maximum value of Youden’s index (YI = specificity + sensitivity − 1). All measurements were carried out in triplicates, and the mean value of replicates, standard deviations, and standard errors from the mean were used to report the results. The statistically significant difference between the binding to MPT64, ESAT-6, and CFP-10, and the difference between the incubation times 5–10 min and 15–20 min was done using one-way analysis of variance analysis.

## Conclusion

This work presented results on the development of the aptasensor for the detection of *M*. *tuberculosis* secreted protein MPT64. The bio receptor – aptamer was previously selected against to MPT64 with disassociation constant *K*_D  _ = 8.92 nM^11^. The aptamers were immobilized on an IDE electrodes and EIS was used as a detection method. The sensitivity of the sensor in buffer was improved down to LoD 4.1 fM compared to previously published data. The incubation time was optimized and set as 15–20 min. The developed aptasensor was tested on clinical samples from TB(+) and TB(−) patients with specificity and sensitivity for the sputum sample analysis 100% and 76.47%, respectively, and for the serum sample analysis 100% and 88.24%, respectively. The proposed aptasensor could be studied and improved further such as analyzing the IDE surface composition after the clinical sample incubation with a spectroscopic technique as well as establishing a threshold for the noise to signal ratio. Overall, this study demonstrated that aptasensor platforms could be successfully employed in clinical sample analyses, aiming to exploit the advantages of this method for biomolecule detection.

## References

[1] Organization, W. H.O.Global tuberculosis report (2018).

[2] Organization, W. H.O. Global tuberculosis report (2017).

[3] Organization, W. H.O Global tuberculosis report (2016).

[4] Lu, J. *et al*. Direct detection from clinical sputum samples to differentiate live and dead Mycobacterium Tuberculosis. *J Clin Lab Anal* **33**, e22716 (2019). 10.1002/jcla.22716 PMC6818545 30461054

[5] Kruh-Garcia Nicole A., Wolfe Lisa M., Chaisson Lelia H., Worodria William O., Nahid Payam, Schorey Jeff S., Davis J. Lucian, Dobos Karen M. (2014). Detection of Mycobacterium tuberculosis Peptides in the Exosomes of Patients with Active and Latent M. tuberculosis Infection Using MRM-MS. PLoS ONE.

[6] Baltzell K (2011). Limited evidence of human papillomavirus on breast tissue using molecular *in situ* methods. Cancer.

[7] Wang Z (2007). The solution structure of antigen MPT64 from Mycobacterium tuberculosis defines a new family of beta-grasp proteins. J. Mol. Biol..

[8] Gaillard T (2011). Assessment of the SD Bioline Ag MPT64 Rapid^TM^ and the MGIT^TM^ TBc identification tests for the diagnosis of tuberculosis. Diagn. Microbiol. Infect. Dis..

[9] Arora, J. *et al*. Utility of MPT64 Antigen Detection for Rapid Confirmation of Mycobacterium tuberculosis Complex. J. Glob. Infect. Dis. **7**, 66–9 (2015). 10.4103/0974-777X.154443 PMC4448327 26069425

[10] Mehaffy, C., Dobos, K. M., Nahid, P. & Kruh-Garcia, N. A. Second generation multiple reaction monitoring assays for enhanced detection of ultra-low abundance Mycobacterium tuberculosis peptides in human serum. *Clin*. *Proteomics* **14** (2017). 10.1186/s12014-017-9156-y PMC5460347 28592925

[11] Sypabekova Marzhan, Bekmurzayeva Aliya, Wang Ronghui, Li Yanbin, Nogues Claude, Kanayeva Damira (2017). Selection, characterization, and application of DNA aptamers for detection of Mycobacterium tuberculosis secreted protein MPT64. Tuberculosis.

[12] Kanayeva, D., Sypabekova, M., Bekmurzayeva, A. & Li, Y. Aptamers for determining bacterial cell Mycobacterium tuberculosis based on secreted protein MPT64, composition containing DNA aptamer and method of detecting mycobacteria in a clinical sample. IPC No. C12P 19/34 (2006.01), C12Q 1/00 (2006.01), Ministry of Justice of the Republic of Kazakhstan, Patent No. 33639, Oct 30 2017 (2019).

[13] Sypabekova, M., Jolly, P., Estrela, P. & Kanayeva, D. Electrochemical Aptasensor using Optimized Surface Chemistry for the Detection of Mycobacterium tuberculosis Secreted Protein MPT64 in Human Serum. *Biosens*. *Bioelectron* (2018). 10.1016/j.bios.2018.07.053 30078622

[14] Dimaki M (2014). A compact microelectrode array chip with multiple measuring sites for electrochemical applications. Sensors.

[15] Armbruster DA, Pry T (2008). Limit of Blank, Limit of Detection and Limit of Quantitation. Clin. Biochem. Rev..

[16] Armbruster DA, Tillman MD, Hubbs LM (1994). Limit of detection (LQD)/limit of quantitation (LOQ): comparison of the empirical and the statistical methods exemplified with GC-MS assays of abused drugs. Clin Chem.

[17] Santos, A., Davis, J. J. & Bueno, P. R. Fundamentals and applications of impedimetric and redox capacitive biosensors. *J*. *Anal*. *Bioanal*. *Tech*. **1** (2015).

[18] Bertok T (2013). Label-free detection of glycoproteins by the lectin biosensor down to attomolar level using gold nanoparticles. Talanta.

[19] Jolly P (2016). DNA aptamer-based sandwich microfluidic assays for dual quantification and multi-glycan profiling of cancer biomarkers. Biosens Bioelectron.

[20] MacKay S, Hermansen P, Wishart D, Chen J (2015). Simulations of interdigitated electrode interactions with gold nanoparticles for impedance-based biosensing applications. Sensors.

[21] Min J, Baeumner AJ (2004). Characterization and optimization of interdigitated ultramicroelectrode arrays as electrochemical biosensor transducers. Electroanal. An Int. J. Devoted to Fundam. Pract. Asp. Electroanal..

[22] Wang R (2015). A label-free impedance immunosensor using screen-printed interdigitated electrodes and magnetic nanobeads for the detection of *E*. *coli* O157: H7. Biosensors.

[23] Lin J (2015). An impedance immunosensor based on low-cost microelectrodes and specific monoclonal antibodies for rapid detection of avian influenza virus H5N1 in chicken swabs. Biosens. Bioelectron..

[24] Zhurauski P (2018). Sensitive and selective Affimer-functionalised interdigitated electrode-based capacitive biosensor for Her4 protein tumour biomarker detection. Biosens. Bioelectron..

[25] Bonanni A (2010). DNA hybridization detection by electrochemical impedance spectroscopy using interdigitated gold nanoelectrodes. Microchim. Acta.

[26] Jolly P (2016). Electro-Engineered Polymeric Films for the Development of Sensitive Aptasensors for Prostate Cancer Marker Detection. ACS Sensors.

[27] Jolly, P. *et al*. Highly sensitive dual mode electrochemical platform for microRNA detection. *Sci*. *Rep*. **6** (2016). 10.1038/srep36719 PMC5099858 27824137

[28] Asad-Ur-Rahman, F. N. U. & Saif, M. W. Elevated level of serum carcinoembryonic antigen (CEA) and search for a malignancy: a case report. *Cureus* **8** (2016). 10.7759/cureus.648 PMC4954749 27446768

[29] Thompson IM (2004). Prevalence of prostate cancer among men with a prostate-specific antigen level≤ 4.0 ng per milliliter. N. Engl. J. Med..

[30] Bekmurzayeva, A., Sypabekova, M. & Kanayeva, D. Tuberculosis diagnosis using immunodominant, secreted antigens of Mycobacterium tuberculosis. *Tuberculosis* **93** (2013). 10.1016/j.tube.2013.03.003 23602700

[31] Renshaw PS (2005). Structure and function of the complex formed by the tuberculosis virulence factors CFP-10 and ESAT-6. EMBO J..

[32] Lightbody KL (2008). Molecular features governing the stability and specificity of functional complex formation by Mycobacterium tuberculosis CFP-10/ESAT-6 family proteins. J. Biol. Chem..

[33] Abebe F, Belay M, Legesse M, Mihret A, Franken KS (2017). Association of ESAT‐6/CFP‐10‐induced IFN‐γ, TNF‐α and IL‐10 with clinical tuberculosis: evidence from cohorts of pulmonary tuberculosis patients, household contacts and community controls in an endemic setting. Clin. Exp. Immunol..

[34] Roche PW, Winter N, Triccas JA, Feng CG, Britton WJ (1996). Expression of Mycobacterium tuberculosis MPT64 in recombinant Myco. smegmatis: purification, immunogenicity and application to skin tests for tuberculosis. Clin. Exp. Immunol..

[35] Rotherham LS, Maserumule C, Dheda K, Theron J, Khati M (2012). Selection and application of ssDNA aptamers to detect active TB from sputum samples. PLoS One.

[36] Keighley SD, Li P, Estrela P, Migliorato P (2008). Optimization of DNA immobilization on gold electrodes for label-free detection by electrochemical impedance spectroscopy. Biosens Bioelectron.

[37] Jolly P (2015). Label-free impedimetric aptasensor with antifouling surface chemistry: A prostate specific antigen case study. Sensors Actuators B Chem..

